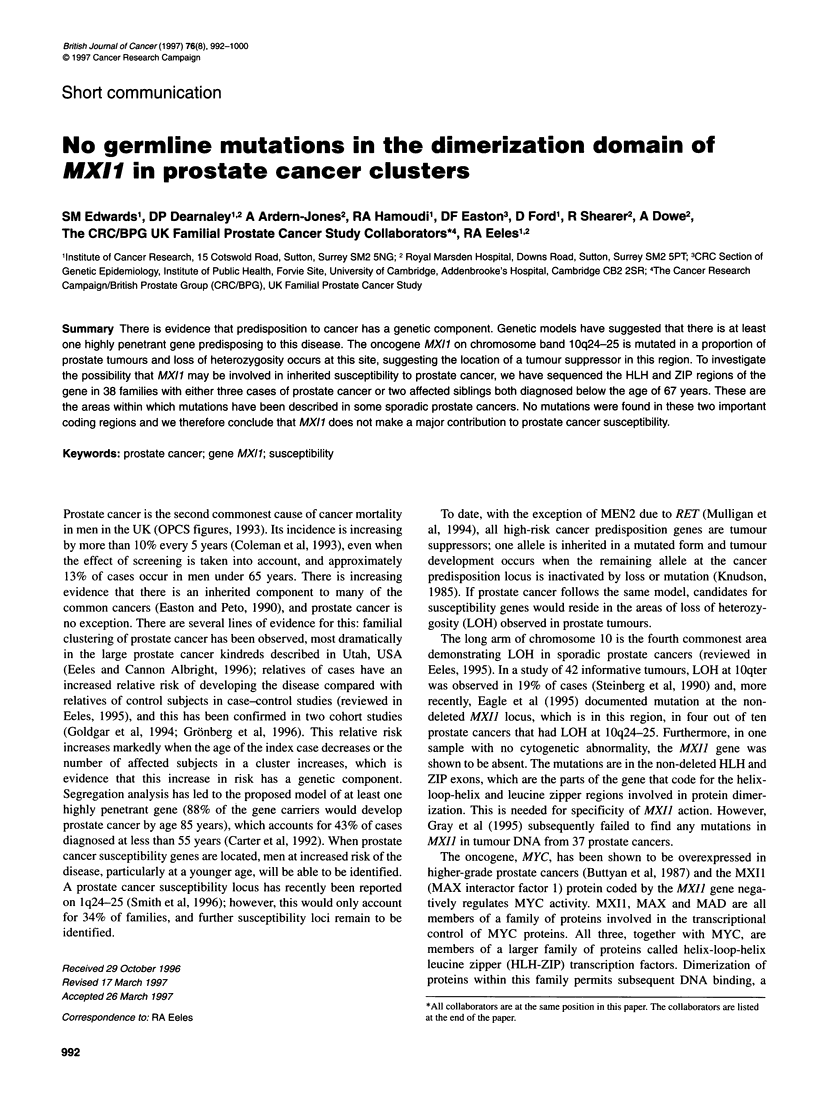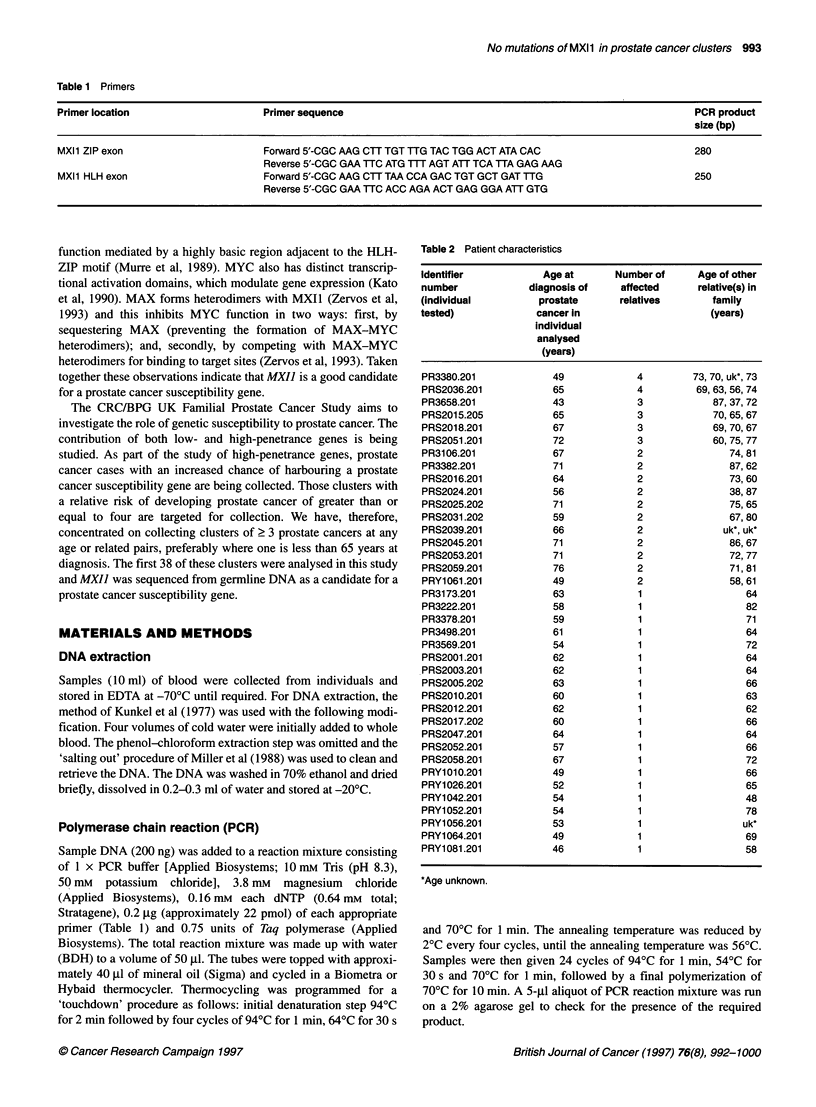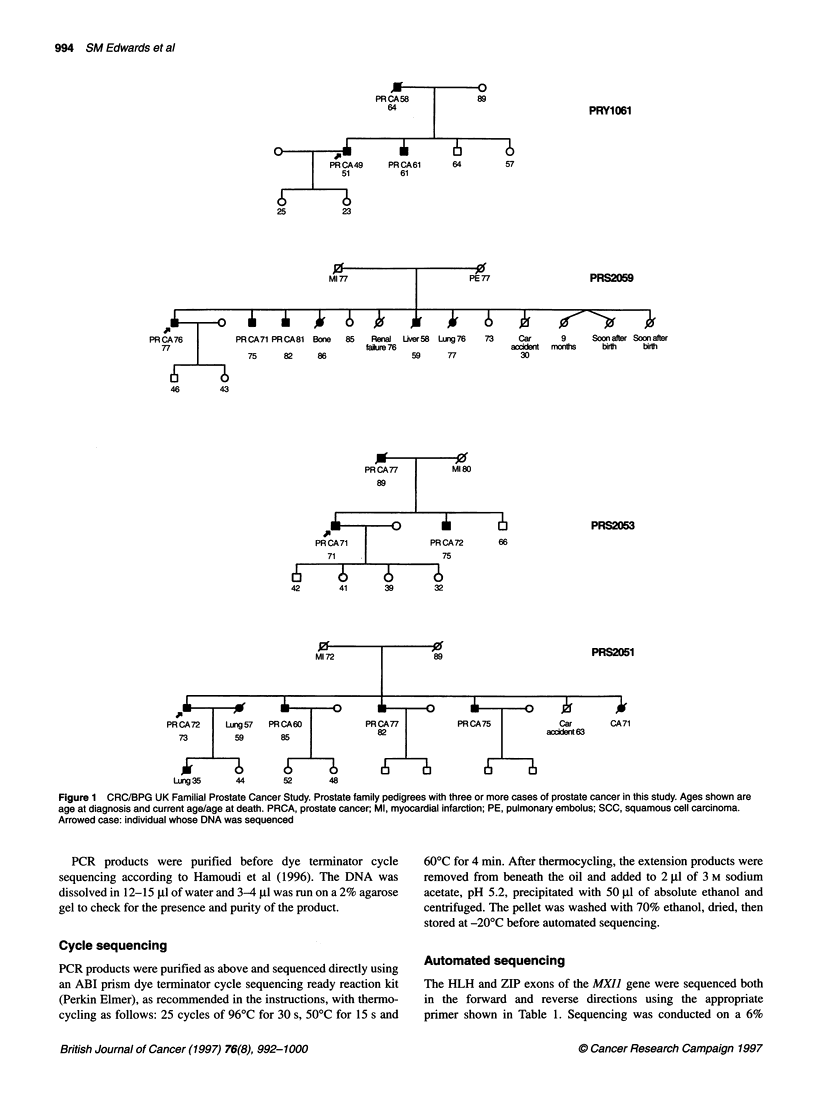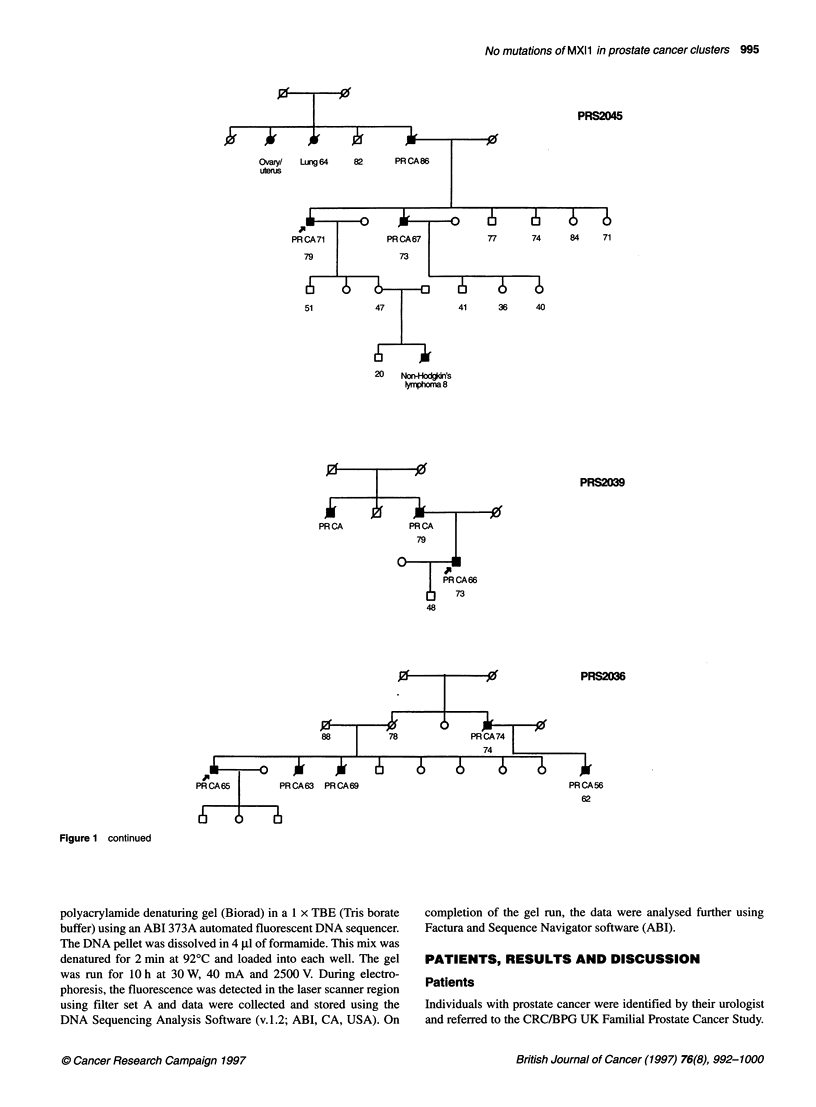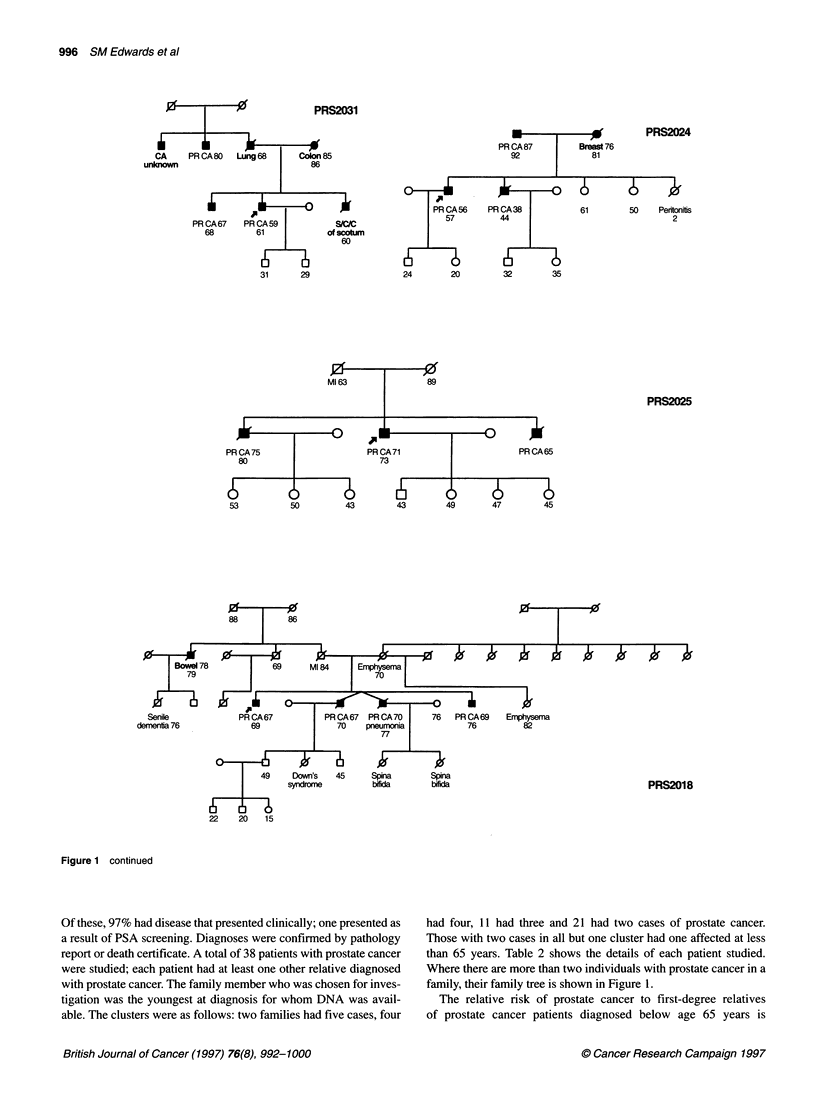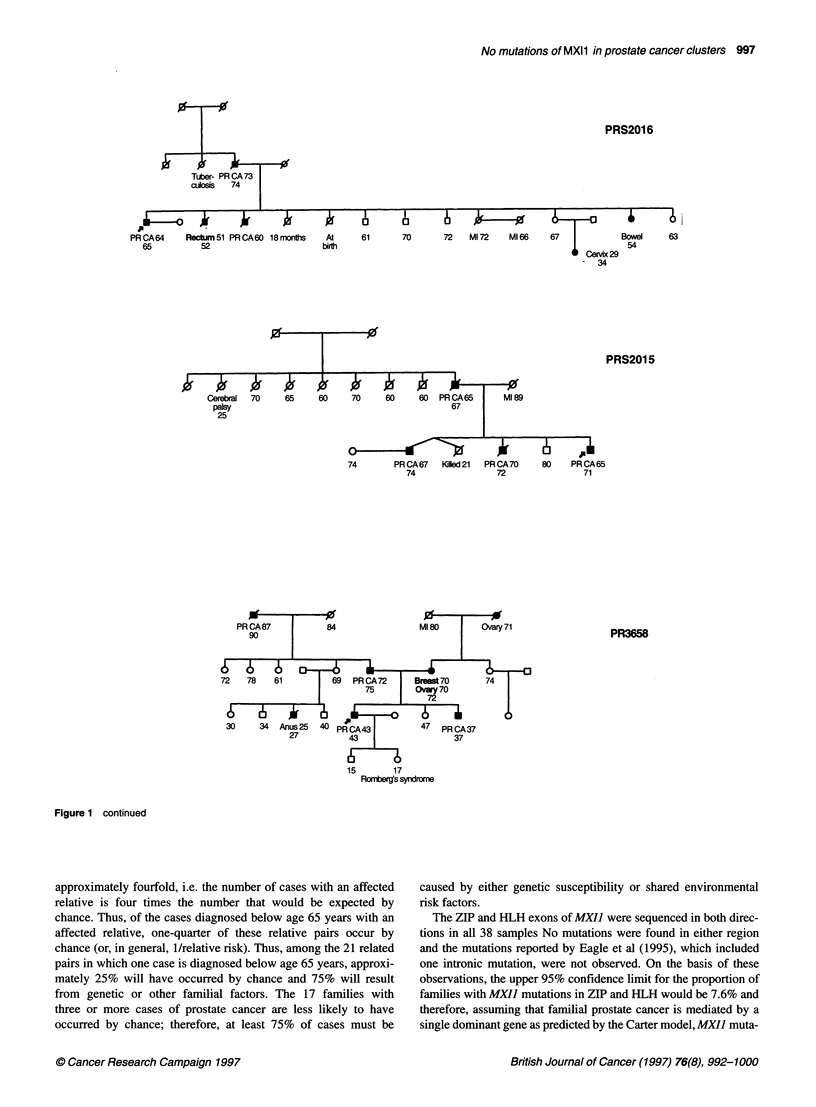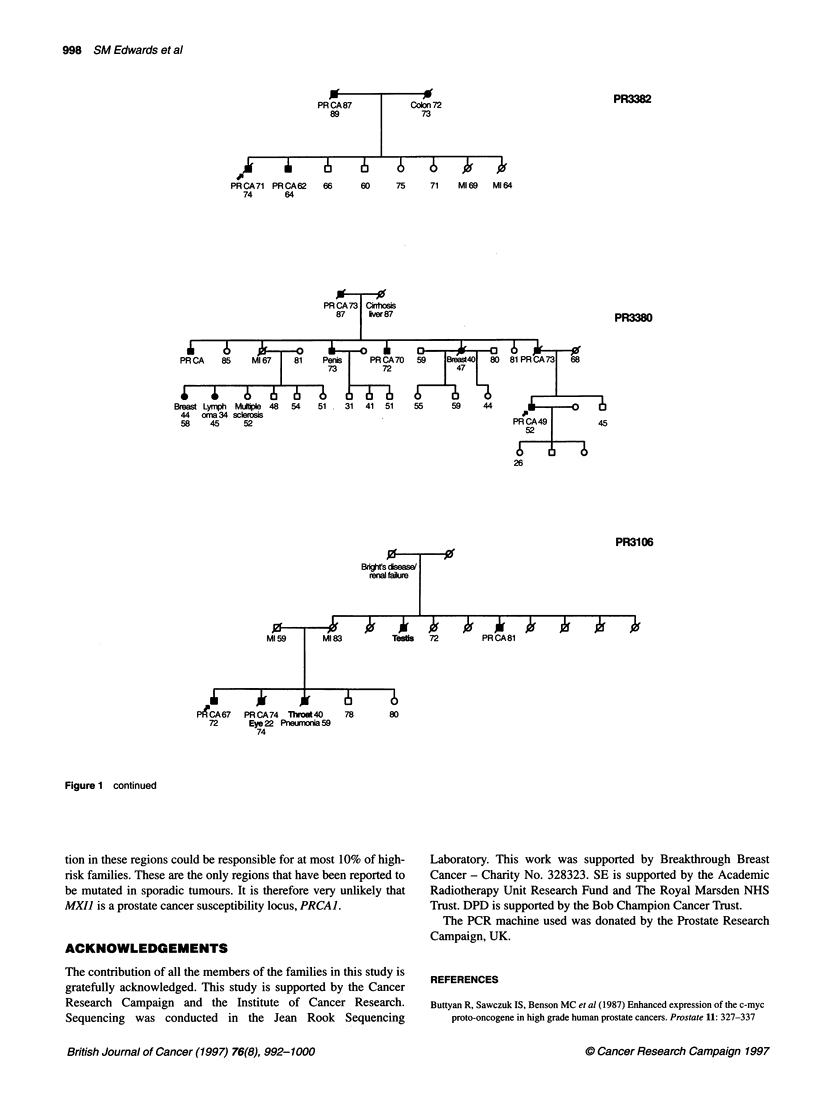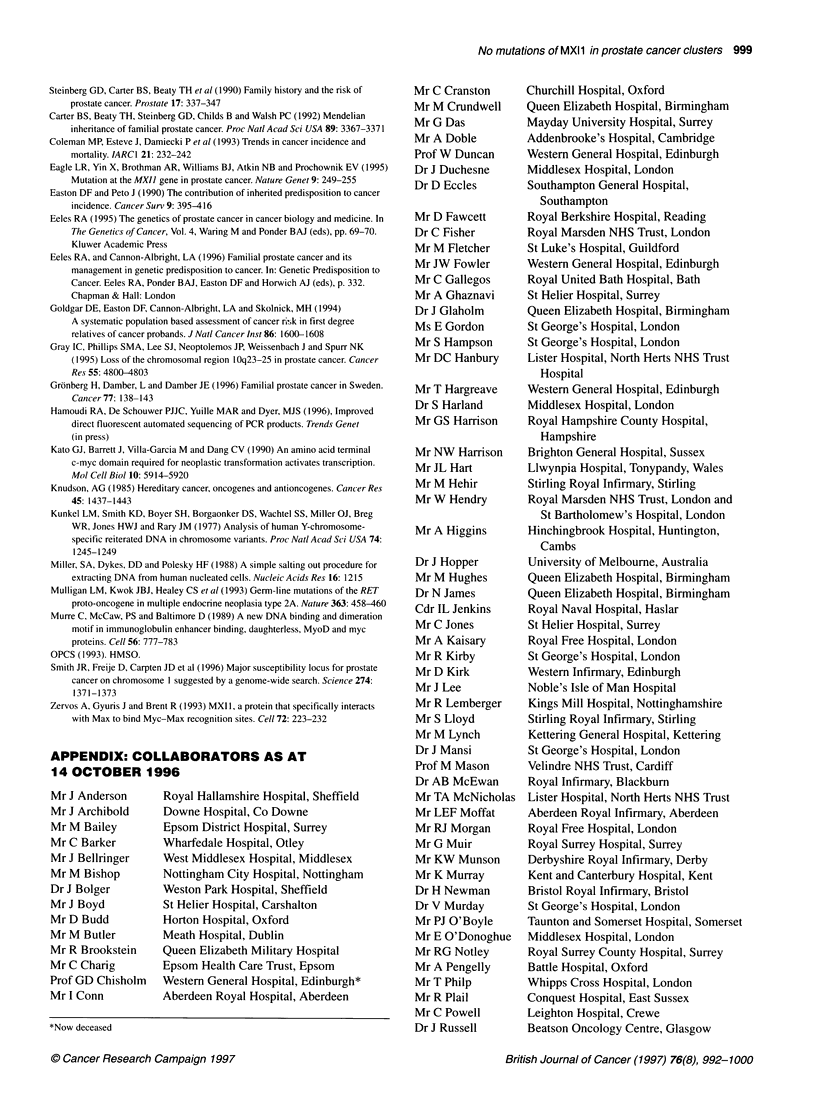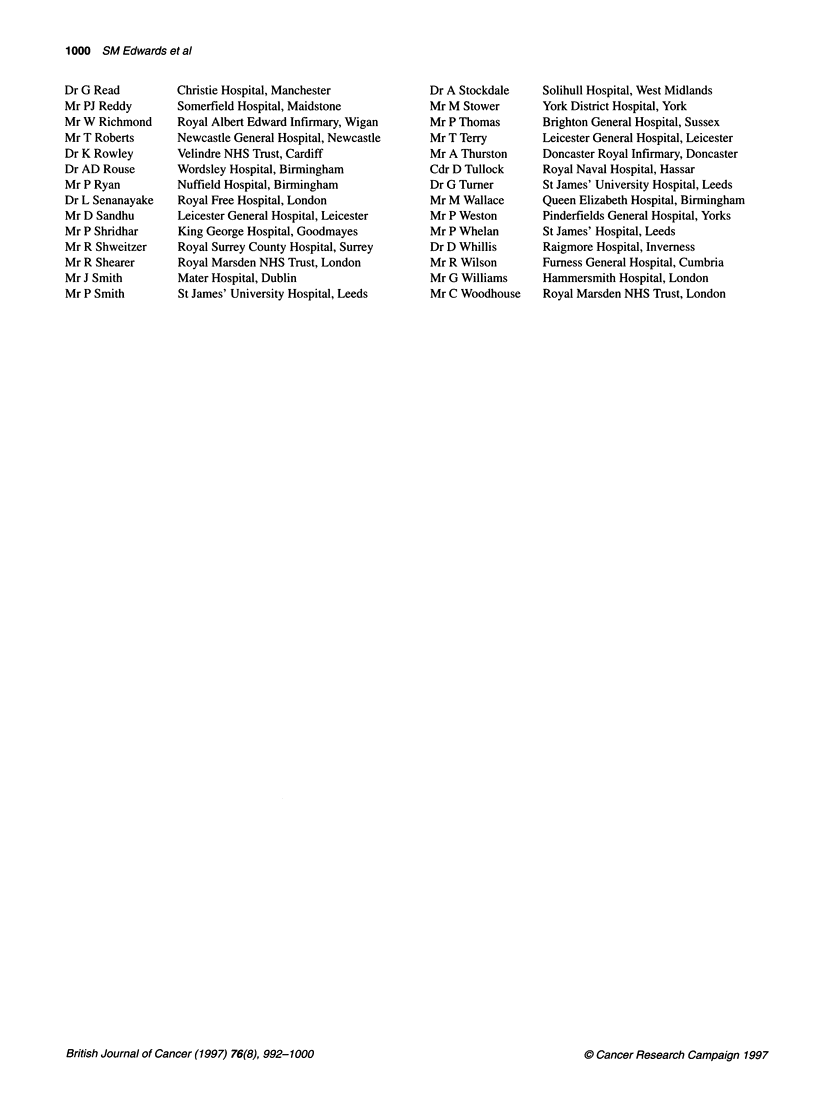# No germline mutations in the dimerization domain of MXI1 in prostate cancer clusters. The CRC/BPG UK Familial Prostate Cancer Study Collaborators. Cancer Research Campaign/British Prostate Group.

**DOI:** 10.1038/bjc.1997.498

**Published:** 1997

**Authors:** S. M. Edwards, D. P. Dearnaley, A. Ardern-Jones, R. A. Hamoudi, D. F. Easton, D. Ford, R. Shearer, A. Dowe, R. A. Eeles

**Affiliations:** Institute of Cancer Research, Sutton, Surrey.

## Abstract

There is evidence that predisposition to cancer has a genetic component. Genetic models have suggested that there is at least one highly penetrant gene predisposing to this disease. The oncogene MXI1 on chromosome band 10q24-25 is mutated in a proportion of prostate tumours and loss of heterozygosity occurs at this site, suggesting the location of a tumour suppressor in this region. To investigate the possibility that MXI1 may be involved in inherited susceptibility to prostate cancer, we have sequenced the HLH and ZIP regions of the gene in 38 families with either three cases of prostate cancer or two affected siblings both diagnosed below the age of 67 years. These are the areas within which mutations have been described in some sporadic prostate cancers. No mutations were found in these two important coding regions and we therefore conclude that MXI1 does not make a major contribution to prostate cancer susceptibility.


British Journal of Cancer (1997) 76(8), 992–1000
© 1997 Cancer Research Campaign

Short communication

### No germline mutations in the dimerization domain of *MXI1* in prostate cancer clusters

**SM Edwards[1], DP Dearnaley[1,2] A Ardern-Jones[2], RA Hamoudi[1], DF Easton[3], D Ford[1], R Shearer[2], A Dowe[2], The CRC/BPG UK Familial Prostate Cancer Study Collaborators*[4], RA Eeles[1,2]**

[1]Institute of Cancer Research, 15 Cotswold Road, Sutton, Surrey SM2 5NG; [2] Royal Marsden Hospital, Downs Road, Sutton, Surrey SM2 5PT; [3]CRC Section of Genetic Epidemiology, Institute of Public Health, Forvie Site, University of Cambridge, Addenbrooke's Hospital, Cambridge CB2 2SR; [4]The Cancer Research Campaign/British Prostate Group (CRC/BPG), UK Familial Prostate Cancer Study

**Summary** There is evidence that predisposition to cancer has a genetic component. Genetic models have suggested that there is at least one highly penetrant gene predisposing to this disease. The oncogene *MXI1* on chromosome band 10q24–25 is mutated in a proportion of prostate tumours and loss of heterozygosity occurs at this site, suggesting the location of a tumour suppressor in this region. To investigate the possibility that *MXI1* may be involved in inherited susceptibility to prostate cancer, we have sequenced the HLH and ZIP regions of the gene in 38 families with either three cases of prostate cancer or two affected siblings both diagnosed below the age of 67 years. These are the areas within which mutations have been described in some sporadic prostate cancers. No mutations were found in these two important coding regions and we therefore conclude that *MXI1* does not make a major contribution to prostate cancer susceptibility.

**Keywords:** prostate cancer; gene *MXI1*; susceptibility

Prostate cancer is the second commonest cause of cancer mortality in men in the UK (OPCS figures, 1993). Its incidence is increasing by more than 10% every 5 years (Coleman et al, 1993), even when the effect of screening is taken into account, and approximately 13% of cases occur in men under 65 years. There is increasing evidence that there is an inherited component to many of the common cancers (Easton and Peto, 1990), and prostate cancer is no exception. There are several lines of evidence for this: familial clustering of prostate cancer has been observed, most dramatically in the large prostate cancer kindreds described in Utah, USA (Eeles and Cannon Albright, 1996); relatives of cases have an increased relative risk of developing the disease compared with relatives of control subjects in case–control studies (reviewed in Eeles, 1995), and this has been confirmed in two cohort studies (Goldgar et al, 1994; Grönberg et al, 1996). This relative risk increases markedly when the age of the index case decreases or the number of affected subjects in a cluster increases, which is evidence that this increase in risk has a genetic component. Segregation analysis has led to the proposed model of at least one highly penetrant gene (88% of the gene carriers would develop prostate cancer by age 85 years), which accounts for 43% of cases diagnosed at less than 55 years (Carter et al, 1992). When prostate cancer susceptibility genes are located, men at increased risk of the disease, particularly at a younger age, will be able to be identified. A prostate cancer susceptibility locus has recently been reported on 1q24–25 (Smith et al, 1996); however, this would only account for 34% of families, and further susceptibility loci remain to be identified.

*Received 29 October 1996*
*Revised 17 March 1997*
*Accepted 26 March 1997*

*Correspondence to:* RA Eeles

To date, with the exception of MEN2 due to *RET* (Mulligan et al, 1994), all high-risk cancer predisposition genes are tumour suppressors; one allele is inherited in a mutated form and tumour development occurs when the remaining allele at the cancer predisposition locus is inactivated by loss or mutation (Knudson, 1985). If prostate cancer follows the same model, candidates for susceptibility genes would reside in the areas of loss of heterozygosity (LOH) observed in prostate tumours.

The long arm of chromosome 10 is the fourth commonest area demonstrating LOH in sporadic prostate cancers (reviewed in Eeles, 1995). In a study of 42 informative tumours, LOH at 10qter was observed in 19% of cases (Steinberg et al, 1990) and, more recently, Eagle et al (1995) documented mutation at the non-deleted *MXI1* locus, which is in this region, in four out of ten prostate cancers that had LOH at 10q24–25. Furthermore, in one sample with no cytogenetic abnormality, the *MXI1* gene was shown to be absent. The mutations are in the non-deleted HLH and ZIP exons, which are the parts of the gene that code for the helix-loop-helix and leucine zipper regions involved in protein dimerization. This is needed for specificity of *MXI1* action. However, Gray et al (1995) subsequently failed to find any mutations in *MXI1* in tumour DNA from 37 prostate cancers.

The oncogene, *MYC*, has been shown to be overexpressed in higher-grade prostate cancers (Buttyan et al, 1987) and the MXI1 (MAX interactor factor 1) protein coded by the *MXI1* gene negatively regulates MYC activity. MXI1, MAX and MAD are all members of a family of proteins involved in the transcriptional control of MYC proteins. All three, together with MYC, are members of a larger family of proteins called helix-loop-helix leucine zipper (HLH-ZIP) transcription factors. Dimerization of proteins within this family permits subsequent DNA binding, a

*All collaborators are at the same position in this paper. The collaborators are listed at the end of the paper.

**Table 1** Primers

| Primer location | Primer sequence | PCR product size (bp) |
|---|---|---|
| MXI1 ZIP exon | Forward 5′-CGC AAG CTT TGT TTG TAC TGG ACT ATA CAC<br>Reverse 5′-CGC GAA TTC ATG TTT AGT ATT TCA TTA GAG AAG | 280 |
| MXI1 HLH exon | Forward 5′-CGC AAG CTT TAA CCA GAC TGT GCT GAT TTG<br>Reverse 5′-CGC GAA TTC ACC AGA ACT GAG GGA ATT GTG | 250 |

function mediated by a highly basic region adjacent to the HLH-ZIP motif (Murre et al, 1989). MYC also has distinct transcriptional activation domains, which modulate gene expression (Kato et al, 1990). MAX forms heterodimers with MXI1 (Zervos et al, 1993) and this inhibits MYC function in two ways: first, by sequestering MAX (preventing the formation of MAX–MYC heterodimers); and, secondly, by competing with MAX–MYC heterodimers for binding to target sites (Zervos et al, 1993). Taken together these observations indicate that *MXI1* is a good candidate for a prostate cancer susceptibility gene.

The CRC/BPG UK Familial Prostate Cancer Study aims to investigate the role of genetic susceptibility to prostate cancer. The contribution of both low- and high-penetrance genes is being studied. As part of the study of high-penetrance genes, prostate cancer cases with an increased chance of harbouring a prostate cancer susceptibility gene are being collected. Those clusters with a relative risk of developing prostate cancer of greater than or equal to four are targeted for collection. We have, therefore, concentrated on collecting clusters of ≥ 3 prostate cancers at any age or related pairs, preferably where one is less than 65 years at diagnosis. The first 38 of these clusters were analysed in this study and *MXI1* was sequenced from germline DNA as a candidate for a prostate cancer susceptibility gene.

**Table 2** Patient characteristics

| Identifier number (individual tested) | Age at diagnosis of prostate cancer in individual analysed (years) | Number of affected relatives | Age of other relative(s) in family (years) |
|---|---|---|---|
| PR3380.201 | 49 | 4 | 73, 70, uk*, 73 |
| PRS2036.201 | 65 | 4 | 69, 63, 56, 74 |
| PR3658.201 | 43 | 3 | 87, 37, 72 |
| PRS2015.205 | 65 | 3 | 70, 65, 67 |
| PRS2018.201 | 67 | 3 | 69, 70, 67 |
| PRS2051.201 | 72 | 3 | 60, 75, 77 |
| PR3106.201 | 67 | 2 | 74, 81 |
| PR3382.201 | 71 | 2 | 87, 62 |
| PRS2016.201 | 64 | 2 | 73, 60 |
| PRS2024.201 | 56 | 2 | 38, 87 |
| PRS2025.202 | 71 | 2 | 75, 65 |
| PRS2031.202 | 59 | 2 | 67, 80 |
| PRS2039.201 | 66 | 2 | uk*, uk* |
| PRS2045.201 | 71 | 2 | 86, 67 |
| PRS2053.201 | 71 | 2 | 72, 77 |
| PRS2059.201 | 76 | 2 | 71, 81 |
| PRY1061.201 | 49 | 2 | 58, 61 |
| PR3173.201 | 63 | 1 | 64 |
| PR3222.201 | 58 | 1 | 82 |
| PR3378.201 | 59 | 1 | 71 |
| PR3498.201 | 61 | 1 | 64 |
| PR3569.201 | 54 | 1 | 72 |
| PRS2001.201 | 62 | 1 | 64 |
| PRS2003.201 | 62 | 1 | 64 |
| PRS2005.202 | 63 | 1 | 66 |
| PRS2010.201 | 60 | 1 | 63 |
| PRS2012.201 | 62 | 1 | 62 |
| PRS2017.202 | 60 | 1 | 66 |
| PRS2047.201 | 64 | 1 | 64 |
| PRS2052.201 | 57 | 1 | 66 |
| PRS2058.201 | 67 | 1 | 72 |
| PRY1010.201 | 49 | 1 | 66 |
| PRY1026.201 | 52 | 1 | 65 |
| PRY1042.201 | 54 | 1 | 48 |
| PRY1052.201 | 54 | 1 | 78 |
| PRY1056.201 | 53 | 1 | uk* |
| PRY1064.201 | 49 | 1 | 69 |
| PRY1081.201 | 46 | 1 | 58 |

*Age unknown.

### MATERIALS AND METHODS

### DNA extraction

Samples (10 ml) of blood were collected from individuals and stored in EDTA at –70°C until required. For DNA extraction, the method of Kunkel et al (1977) was used with the following modification. Four volumes of cold water were initially added to whole blood. The phenol–chloroform extraction step was omitted and the 'salting out' procedure of Miller et al (1988) was used to clean and retrieve the DNA. The DNA was washed in 70% ethanol and dried briefly, dissolved in 0.2–0.3 ml of water and stored at –20°C.

### Polymerase chain reaction (PCR)

Sample DNA (200 ng) was added to a reaction mixture consisting of 1 × PCR buffer [Applied Biosystems; 10 mM Tris (pH 8.3), 50 mM potassium chloride], 3.8 mM magnesium chloride (Applied Biosystems), 0.16 mM each dNTP (0.64 mM total; Stratagene), 0.2 μg (approximately 22 pmol) of each appropriate primer (Table 1) and 0.75 units of *Taq* polymerase (Applied Biosystems). The total reaction mixture was made up with water (BDH) to a volume of 50 μl. The tubes were topped with approximately 40 μl of mineral oil (Sigma) and cycled in a Biometra or Hybaid thermocycler. Thermocycling was programmed for a 'touchdown' procedure as follows: initial denaturation step 94°C for 2 min followed by four cycles of 94°C for 1 min, 64°C for 30 s and 70°C for 1 min. The annealing temperature was reduced by 2°C every four cycles, until the annealing temperature was 56°C. Samples were then given 24 cycles of 94°C for 1 min, 54°C for 30 s and 70°C for 1 min, followed by a final polymerization of 70°C for 10 min. A 5-μl aliquot of PCR reaction mixture was run on a 2% agarose gel to check for the presence of the required product.

© Cancer Research Campaign 1997

**Figure 1** CRC/BPG UK Familial Prostate Cancer Study. Prostate family pedigrees with three or more cases of prostate cancer in this study. Ages shown are age at diagnosis and current age/age at death. PRCA, prostate cancer; MI, myocardial infarction; PE, pulmonary embolus; SCC, squamous cell carcinoma. Arrowed case: individual whose DNA was sequenced

PCR products were purified before dye terminator cycle sequencing according to Hamoudi et al (1996). The DNA was dissolved in 12–15 µl of water and 3–4 µl was run on a 2% agarose gel to check for the presence and purity of the product.

### Cycle sequencing

PCR products were purified as above and sequenced directly using an ABI prism dye terminator cycle sequencing ready reaction kit (Perkin Elmer), as recommended in the instructions, with thermocycling as follows: 25 cycles of 96°C for 30 s, 50°C for 15 s and 60°C for 4 min. After thermocycling, the extension products were removed from beneath the oil and added to 2 µl of 3 M sodium acetate, pH 5.2, precipitated with 50 µl of absolute ethanol and centrifuged. The pellet was washed with 70% ethanol, dried, then stored at –20°C before automated sequencing.

### Automated sequencing

The HLH and ZIP exons of the *MXI1* gene were sequenced both in the forward and reverse directions using the appropriate primer shown in Table 1. Sequencing was conducted on a 6%

© Cancer Research Campaign 1997

**Figure 1** continued polyacrylamide denaturing gel (Biorad) in a 1 × TBE (Tris borate buffer) using an ABI 373A automated fluorescent DNA sequencer. The DNA pellet was dissolved in 4 µl of formamide. This mix was denatured for 2 min at 92°C and loaded into each well. The gel was run for 10 h at 30 W, 40 mA and 2500 V. During electrophoresis, the fluorescence was detected in the laser scanner region using filter set A and data were collected and stored using the DNA Sequencing Analysis Software (v.1.2; ABI, CA, USA). On completion of the gel run, the data were analysed further using Factura and Sequence Navigator software (ABI).

### PATIENTS, RESULTS AND DISCUSSION

### Patients

Individuals with prostate cancer were identified by their urologist and referred to the CRC/BPG UK Familial Prostate Cancer Study.

© Cancer Research Campaign 1997

**Figure 1** continued

Of these, 97% had disease that presented clinically; one presented as a result of PSA screening. Diagnoses were confirmed by pathology report or death certificate. A total of 38 patients with prostate cancer were studied; each patient had at least one other relative diagnosed with prostate cancer. The family member who was chosen for investigation was the youngest at diagnosis for whom DNA was available. The clusters were as follows: two families had five cases, four had four, 11 had three and 21 had two cases of prostate cancer. Those with two cases in all but one cluster had one affected at less than 65 years. Table 2 shows the details of each patient studied. Where there are more than two individuals with prostate cancer in a family, their family tree is shown in Figure 1.

The relative risk of prostate cancer to first-degree relatives of prostate cancer patients diagnosed below age 65 years is

© Cancer Research Campaign 1997

**Figure 1** continued approximately fourfold, i.e. the number of cases with an affected relative is four times the number that would be expected by chance. Thus, of the cases diagnosed below age 65 years with an affected relative, one-quarter of these relative pairs occur by chance (or, in general, 1/relative risk). Thus, among the 21 related pairs in which one case is diagnosed below age 65 years, approximately 25% will have occurred by chance and 75% will result from genetic or other familial factors. The 17 families with three or more cases of prostate cancer are less likely to have occurred by chance; therefore, at least 75% of cases must be caused by either genetic susceptibility or shared environmental risk factors.

The ZIP and HLH exons of *MXI1* were sequenced in both directions in all 38 samples No mutations were found in either region and the mutations reported by Eagle et al (1995), which included one intronic mutation, were not observed. On the basis of these observations, the upper 95% confidence limit for the proportion of families with *MXI1* mutations in ZIP and HLH would be 7.6% and therefore, assuming that familial prostate cancer is mediated by a single dominant gene as predicted by the Carter model, *MXI1* muta-

**Figure 1** continued tion in these regions could be responsible for at most 10% of high-risk families. These are the only regions that have been reported to be mutated in sporadic tumours. It is therefore very unlikely that *MXI1* is a prostate cancer susceptibility locus, *PRCA1*.

### ACKNOWLEDGEMENTS

The contribution of all the members of the families in this study is gratefully acknowledged. This study is supported by the Cancer Research Campaign and the Institute of Cancer Research. Sequencing was conducted in the Jean Rook Sequencing Laboratory. This work was supported by Breakthrough Breast Cancer – Charity No. 328323. SE is supported by the Academic Radiotherapy Unit Research Fund and The Royal Marsden NHS Trust. DPD is supported by the Bob Champion Cancer Trust.

The PCR machine used was donated by the Prostate Research Campaign, UK.

### REFERENCES

Buttyan R, Sawczuk IS, Benson MC *et al* (1987) Enhanced expression of the c-myc proto-oncogene in high grade human prostate cancers. *Prostate* **11**: 327–337

© Cancer Research Campaign 1997

Steinberg GD, Carter BS, Beaty TH *et al* (1990) Family history and the risk of prostate cancer. *Prostate* **17**: 337–347

Carter BS, Beaty TH, Steinberg GD, Childs B and Walsh PC (1992) Mendelian inheritance of familial prostate cancer. *Proc Natl Acad Sci USA* **89**: 3367–3371

Coleman MP, Esteve J, Damiecki P *et al* (1993) Trends in cancer incidence and mortality. *IARC*1 **21**: 232–242

Eagle LR, Yin X, Brothman AR, Williams BJ, Atkin NB and Prochownik EV (1995) Mutation at the *MXI1* gene in prostate cancer. *Nature Genet* **9**: 249–255

Easton DF and Peto J (1990) The contribution of inherited predisposition to cancer incidence. *Cancer Surv* **9**: 395–416

Eeles RA (1995) The genetics of prostate cancer in cancer biology and medicine. In *The Genetics of Cancer*, Vol. 4, Waring M and Ponder BAJ (eds), pp. 69–70. Kluwer Academic Press

Eeles RA, and Cannon-Albright, LA (1996) Familial prostate cancer and its management in genetic predisposition to cancer. In: Genetic Predisposition to Cancer. Eeles RA, Ponder BAJ, Easton DF and Horwich AJ (eds), p. 332. Chapman & Hall: London

Goldgar DE, Easton DF, Cannon-Albright, LA and Skolnick, MH (1994) A systematic population based assessment of cancer risk in first degree relatives of cancer probands. *J Natl Cancer Inst* **86**: 1600–1608

Gray IC, Phillips SMA, Lee SJ, Neoptolemos JP, Weissenbach J and Spurr NK (1995) Loss of the chromosomal region 10q23–25 in prostate cancer. *Cancer Res* **55**: 4800–4803

Grönberg H, Damber, L and Damber JE (1996) Familial prostate cancer in Sweden. *Cancer* **77**: 138–143

Hamoudi RA, De Schouwer PJJC, Yuille MAR and Dyer, MJS (1996), Improved direct fluorescent automated sequencing of PCR products. *Trends Genet* (in press)

Kato GJ, Barrett J, Villa-Garcia M and Dang CV (1990) An amino acid terminal c-myc domain required for neoplastic transformation activates transcription. *Mol Cell Biol* **10**: 5914–5920

Knudson, AG (1985) Hereditary cancer, oncogenes and antioncogenes. *Cancer Res* **45**: 1437–1443

Kunkel LM, Smith KD, Boyer SH, Borgaonker DS, Wachtel SS, Miller OJ, Breg WR, Jones HWJ and Rary JM (1977) Analysis of human Y-chromosome-specific reiterated DNA in chromosome variants. *Proc Natl Acad Sci USA* **74**: 1245–1249

Miller, SA, Dykes, DD and Polesky HF (1988) A simple salting out procedure for extracting DNA from human nucleated cells. *Nucleic Acids Res* **16**: 1215

Mulligan LM, Kwok JBJ, Healey CS *et al* (1993) Germ-line mutations of the *RET* proto-oncogene in multiple endocrine neoplasia type 2A. *Nature* **363**: 458–460

Murre C, McCaw, PS and Baltimore D (1989) A new DNA binding and dimeration motif in immunoglobulin enhancer binding, daughterless, MyoD and myc proteins. *Cell* **56**: 777–783

OPCS (1993). HMSO.

Smith JR, Freije D, Carpten JD et al (1996) Major susceptibility locus for prostate cancer on chromosome 1 suggested by a genome-wide search. *Science* **274**: 1371–1373

Zervos A, Gyuris J and Brent R (1993) MXI1, a protein that specifically interacts with Max to bind Myc–Max recognition sites. *Cell* **72**: 223–232

### APPENDIX: COLLABORATORS AS AT 14 OCTOBER 1996

| | |
|---|---|
| Mr J Anderson | Royal Hallamshire Hospital, Sheffield |
| Mr J Archibold | Downe Hospital, Co Downe |
| Mr M Bailey | Epsom District Hospital, Surrey |
| Mr C Barker | Wharfedale Hospital, Otley |
| Mr J Bellringer | West Middlesex Hospital, Middlesex |
| Mr M Bishop | Nottingham City Hospital, Nottingham |
| Dr J Bolger | Weston Park Hospital, Sheffield |
| Mr J Boyd | St Helier Hospital, Carshalton |
| Mr D Budd | Horton Hospital, Oxford |
| Mr M Butler | Meath Hospital, Dublin |
| Mr R Brookstein | Queen Elizabeth Military Hospital |
| Mr C Charig | Epsom Health Care Trust, Epsom |
| Prof GD Chisholm | Western General Hospital, Edinburgh* |
| Mr I Conn | Aberdeen Royal Hospital, Aberdeen |
| Mr C Cranston | Churchill Hospital, Oxford |
| Mr M Crundwell | Queen Elizabeth Hospital, Birmingham |
| Mr G Das | Mayday University Hospital, Surrey |
| Mr A Doble | Addenbrooke's Hospital, Cambridge |
| Prof W Duncan | Western General Hospital, Edinburgh |
| Dr J Duchesne | Middlesex Hospital, London |
| Dr D Eccles | Southampton General Hospital, Southampton |
| Mr D Fawcett | Royal Berkshire Hospital, Reading |
| Dr C Fisher | Royal Marsden NHS Trust, London |
| Mr M Fletcher | St Luke's Hospital, Guildford |
| Mr JW Fowler | Western General Hospital, Edinburgh |
| Mr C Gallegos | Royal United Bath Hospital, Bath |
| Mr A Ghaznavi | St Helier Hospital, Surrey |
| Dr J Glaholm | Queen Elizabeth Hospital, Birmingham |
| Ms E Gordon | St George's Hospital, London |
| Mr S Hampson | St George's Hospital, London |
| Mr DC Hanbury | Lister Hospital, North Herts NHS Trust Hospital |
| Mr T Hargreave | Western General Hospital, Edinburgh |
| Dr S Harland | Middlesex Hospital, London |
| Mr GS Harrison | Royal Hampshire County Hospital, Hampshire |
| Mr NW Harrison | Brighton General Hospital, Sussex |
| Mr JL Hart | Llwynpia Hospital, Tonypandy, Wales |
| Mr M Hehir | Stirling Royal Infirmary, Stirling |
| Mr W Hendry | Royal Marsden NHS Trust, London and St Bartholomew's Hospital, London |
| Mr A Higgins | Hinchingbrook Hospital, Huntington, Cambs |
| Dr J Hopper | University of Melbourne, Australia |
| Mr M Hughes | Queen Elizabeth Hospital, Birmingham |
| Dr N James | Queen Elizabeth Hospital, Birmingham |
| Cdr IL Jenkins | Royal Naval Hospital, Haslar |
| Mr C Jones | St Helier Hospital, Surrey |
| Mr A Kaisary | Royal Free Hospital, London |
| Mr R Kirby | St George's Hospital, London |
| Mr D Kirk | Western Infirmary, Edinburgh |
| Mr J Lee | Noble's Isle of Man Hospital |
| Mr R Lemberger | Kings Mill Hospital, Nottinghamshire |
| Mr S Lloyd | Stirling Royal Infirmary, Stirling |
| Mr M Lynch | Kettering General Hospital, Kettering |
| Dr J Mansi | St George's Hospital, London |
| Prof M Mason | Velindre NHS Trust, Cardiff |
| Dr AB McEwan | Royal Infirmary, Blackburn |
| Mr TA McNicholas | Lister Hospital, North Herts NHS Trust |
| Mr LEF Moffat | Aberdeen Royal Infirmary, Aberdeen |
| Mr RJ Morgan | Royal Free Hospital, London |
| Mr G Muir | Royal Surrey Hospital, Surrey |
| Mr KW Munson | Derbyshire Royal Infirmary, Derby |
| Mr K Murray | Kent and Canterbury Hospital, Kent |
| Dr H Newman | Bristol Royal Infirmary, Bristol |
| Dr V Murday | St George's Hospital, London |
| Mr PJ O'Boyle | Taunton and Somerset Hospital, Somerset |
| Mr E O'Donoghue | Middlesex Hospital, London |
| Mr RG Notley | Royal Surrey County Hospital, Surrey |
| Mr A Pengelly | Battle Hospital, Oxford |
| Mr T Philp | Whipps Cross Hospital, London |
| Mr R Plail | Conquest Hospital, East Sussex |
| Mr C Powell | Leighton Hospital, Crewe |
| Dr J Russell | Beatson Oncology Centre, Glasgow |

*Now deceased

| | |
|---|---|
| Dr G Read | Christie Hospital, Manchester |
| Mr PJ Reddy | Somerfield Hospital, Maidstone |
| Mr W Richmond | Royal Albert Edward Infirmary, Wigan |
| Mr T Roberts | Newcastle General Hospital, Newcastle |
| Dr K Rowley | Velindre NHS Trust, Cardiff |
| Dr AD Rouse | Wordsley Hospital, Birmingham |
| Mr P Ryan | Nuffield Hospital, Birmingham |
| Dr L Senanayake | Royal Free Hospital, London |
| Mr D Sandhu | Leicester General Hospital, Leicester |
| Mr P Shridhar | King George Hospital, Goodmayes |
| Mr R Shweitzer | Royal Surrey County Hospital, Surrey |
| Mr R Shearer | Royal Marsden NHS Trust, London |
| Mr J Smith | Mater Hospital, Dublin |
| Mr P Smith | St James' University Hospital, Leeds |
| Dr A Stockdale | Solihull Hospital, West Midlands |
| Mr M Stower | York District Hospital, York |
| Mr P Thomas | Brighton General Hospital, Sussex |
| Mr T Terry | Leicester General Hospital, Leicester |
| Mr A Thurston | Doncaster Royal Infirmary, Doncaster |
| Cdr D Tullock | Royal Naval Hospital, Hassar |
| Dr G Turner | St James' University Hospital, Leeds |
| Mr M Wallace | Queen Elizabeth Hospital, Birmingham |
| Mr P Weston | Pinderfields General Hospital, Yorks |
| Mr P Whelan | St James' Hospital, Leeds |
| Dr D Whillis | Raigmore Hospital, Inverness |
| Mr R Wilson | Furness General Hospital, Cumbria |
| Mr G Williams | Hammersmith Hospital, London |
| Mr C Woodhouse | Royal Marsden NHS Trust, London |

*© Cancer Research Campaign 1997*